# Oral versus Vaginal Micronized Progesterone for the Treatment of Threatened Miscarriage

**DOI:** 10.12669/pjms.37.3.3700

**Published:** 2021

**Authors:** Rashida Parveen, Mehnaz Khakwani, Sobia Tabassum, Sajjad Masood

**Affiliations:** 1 Rashida Parveen, FCPS (OBG). Department of Obstetrics and Gyne, Unit-II, Nishtar Medical University Hospital, Multan, Pakistan; 2 Mehnaz Khakwani, FCPS (OBG). Department of Obstetrics and Gyne, Unit-II, Nishtar Medical University Hospital, Multan, Pakistan; 3 Sobia Tabassum, FCPS (OBG). Department of Obstetrics and Gyne, Civil Hospital, Bahawalpur, Pakistan; 4 Sajjad Masood, FCPS (OBG). Department of Obstetrics and Gyne, Unit-II, Nishtar Medical University Hospital, Multan, Pakistan

**Keywords:** Gestational age, Parity, Progesterone, Threatened miscarriage, Vaginal bleeding

## Abstract

**Objectives:**

This study was planned with an aim to find out the effectiveness of oral versus vaginal micronized progesterone for the treatment of threatened miscarriage.

**Methods:**

This randomized controlled trial was conducted at The Department of Obstetrics and Gynaecology, Nishtar Hospital Multan, from August 2019 to January 2020. A total of 136 pregnant women, aged 18 to 45 years having vaginal bleeding were included and divided into two groups (68 women in each group). Participants in the Group-A were given oral micronized progesterone as 200mg twice a day while Group-B participants were given vaginal progesterone suppository 400mg once a day. All women were followed up until 20^th^ week of their pregnancy. Outcome was labeled as prevention of miscarriage if woman had no bleeding per vagina and pregnancy went beyond 20^th^ weeks of gestation.

**Results:**

In a total of 136 women enrolled, mean age was noted to be 30.85+3.34 years. Overall, mean gestational age was noted to be 9.3+2.7 weeks. A total of 98 women (49 in each group) completed the follow up and were included in the final analysis regarding outcome. Among Groups-A, 45 (91.8) had prevention of miscarriage while 4 (9.2%) had miscarriage in comparison to 36 (73.5%) in Group-B had prevention of miscarriage whereas 13 (26.5%) had miscarriage and this difference was statistically significant in between the both study groups as women in Group-A had significantly better outcome in terms of prevention of miscarriage. (P value = 0.0164).

**Conclusion:**

The use of oral micronized progesterone was found to be significantly more effective than vaginal progesterone in women with threatened miscarriage.

## INTRODUCTION

Miscarriage is described as loss of spontaneous pregnancy prior 20 weeks of gestation.^1^ Chromosomal anomalies, implantation malfunction and clinical miscarriages are some of the most common reasons for miscarriages.^2^ Recent decades have seen lots of advancements for prevention and management of women who are at risk of clinical miscarriage at the early phase of their pregnancy but still it is a matter of real concern for the healthcare professionals.

Threatened miscarriage, described as vaginal bleeding in the presence or absence of abdominal cramps, is considered the commonest complication of early pregnancy and estimated to occur in about 20% of pregnancies prior to 20^th^ week of gestation.^3^ Well known risk factors of threatened miscarriage are high maternal age, high body mass index prior to pregnancy and low serum progesterone levels.^4^-^6^

Progesterone is commonly termed as “pregnancy hormone”, has a more improvised role during early pregnancy as it is responsible for the preparation of the endometrium for the implantation as well as maintenance of gestational sac in the uterus.^7^ On the other hand, low levels of serum progesterone have been seen to cause threatened miscarriage. Progesterone is frequently considered as the standard choice for treatment of threatened miscarriage around the world. Researchers have noted progesterone to help in promoting muscle protein synthesis in utero, aid sensitivity of prostaglandin and estrogen as well as playing a major role in preventing early contraction of the myometrium.^8^

Progesterone can be administered as orally, intramuscularly or as vaginal suppository. Oral route of progesterone administration ensures maximum compliance but the efficacy of oral progesterone has been found to have varying results. A Cochrane review in 2018 found oral progesterone to “probably minimize the miscarriage rates” in comparison to no treatment (relative risk 0.57, 95% confidence interval 0.38-0.85) but all the trials included were noted to have moderate quality evidence.^9^ In a local study Abrar S et al noted effectiveness of oral versus vaginal progesterone in terms of absence of bleeding per vagina and pregnancy going beyond 20^th^ week. They noted that oral progesterone group was having an efficacy of 90% in comparison to 71% in the vaginal progesterone group.^10^

Scarcity of local data exists about the role of different preparations of progesterone for the treatment of threatened miscarriage so this study was planned with an aim to find out the effectiveness of oral versus vaginal micronized progesterone for the treatment of threatened miscarriage. As use of oral as well as vaginal progesterone is a common practice but not much is on record about its effectiveness. Likewise, no research has been conducted locally to find out the effectiveness of micronized progesterone for the treatment of threatened miscarriage so the results of this research were thought to provide local evidence to obstetricians commonly handling women with threatened miscarriages.

## METHODS

This randomized controlled trial was conducted in the Department of Obstetrics and Gynaecology, Nishtar Hospital Multan, from July 2019 to January 2020. Approval from Institutional Ethical Committee (Ref. No.15226, Dated 12-08-2020) was taken for this study. Informed consent was taken from all the study participants.

A sample size of 136 women was calculated taking 2-sided significance level as 95%, with power 80%, efficacy of oral progesterone as 90% and vaginal progesterone effectiveness as 71%.^10^ All pregnant women included in this study were aged 18 to 45 years having vaginal bleeding. All included cases were having singleton pregnancy while verification of fetal heart activity as well as gestational age less than 12 weeks was made with the help of ultrasonography. Women with any kind of systemic illness or having fever, history of trauma, or loss of conception tissue, or those with bleeding disorders were not enrolled. Women having any kind of uterine or fetal anomaly were also excluded. Vaginal bleeding was variables as spotting was defined as per World Health Organization’s definition of spotting which is “vaginal bleeding that does not require sanitary protection”. Bleeding more than spotting was considered as moderate.

All women included were randomly divided into two groups (68 in each group). Group-A contained cases who were given oral micronized progesterone as 200mg twice a day while Group-B was given vaginal progesterone suppository 400mg once a day. All women were advised to have rest and maintain hydration. All women were followed up until 20^th^ week of their pregnancy. Data regarding outcome was included of all those pregnant women who completed the follow up until 20^th^ weeks of gestation. Outcome was labeled as prevention of miscarriage if woman had no bleeding per vagina and pregnancy went beyond 20^th^ weeks of gestation.

All the study data was analyzed using SPSS version 26.0. Qualitative variables like parity status, abdominal cramps, gestational age and outcome between both study groups were compared using chi-square test while comparison of quantitative variables like age (years) was made through independent sample t–test. P values less than 0.05 were taken as significant.

## RESULTS

In a total of 136 women enrolled, mean age was noted to be 30.85+3.34 years. Overall, mean gestational age was noted to be 9.3+2.7 weeks. Clinical characteristics of women in both study groups in terms of mean age, parity status, abdominal cramps, vaginal bleeding and gestational age are given in Table-I. No significant statistical difference was observed in between the two study groups.

**Table-I:** Clinical Characteristics of Study Participants among Both Study Groups (n=136).

Characteristics		Group-A (n=68)	Group-B (n=68)	P Value
Age in Years (Mean ± SD)		30.57±3.42	31.14±3.27	0.3223
Gestational Age in Weeks (Mean + SD)		9.2+2.1	9.7+2.2	0.1775
Parity Status	Nulliparous	37 (54.4%)	38 (55.9%)	0.8631
	Multiparous	31 (45.6%)	30 (44.1%)	
Abdominal Cramp	Yes	45 (66.2%)	42 (61.8%)	0.5921
	No	23 (33.8%)	26 (38.2%)	
Vaginal Bleeding Status	Moderate	13 (19.1%)	15 (22.1%)	0.6715
	Spotting	55 (80.9%)	53 (67.9%)	
Gestational Age (weeks)	<6	5 (7.4%)	7 (10.3%)	
	> 6 to 8	25 (36.7%)	22 (32.4%)	0.7642
	> 8 to 12	38 (55.9%)	39 (57.4%)	

The comparison of outcome in between both the study groups is highlighted in Fig-I. A total of 98 women (49 in each group) completed the follow up and were included in the final analysis regarding outcome. Among Groups-A, 45 (91.8) had prevention of miscarriage while 4 (9.2%) had miscarriage in comparison to 36 (73.5%) in Group-B had prevention of miscarriage whereas 13 (26.5%) had miscarriage and this difference was statistically significant in between the both study groups as women in Group-A had significantly better outcome in terms of prevention of miscarriage. (P value = 0.0164).

**Fig.1:**
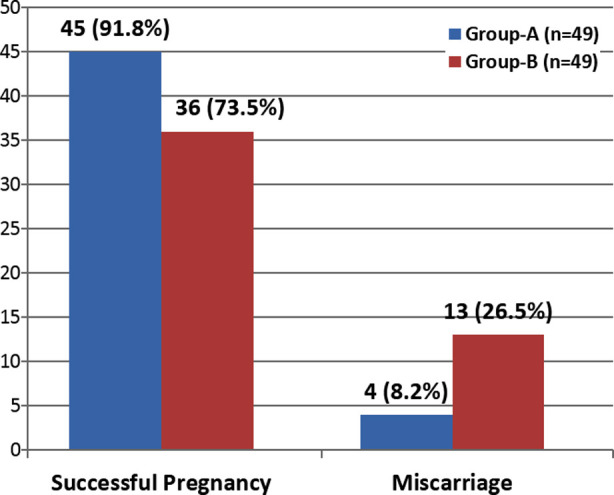
Comparison of Outcome between Oral and Vaginal Micronized Progesterone. P-Value = 0.0164 (significant)

## DISCUSSION

Common symptoms of TM are vaginal bleeding with or without abdominal pain whereas the cervix is closed having alive embryo or fetus inside the womb. Progesterone helps the uterus in preparation regarding implantation of the fertilized egg and suppresses uterine contractions until term.^9^,^11^ Medicines that imitate actions of progesterone are called progestogens. We aimed this study to find out our local experience about whether progesterone is effective among women of threatened miscarriage.

In the present study, it was seen that among women of Group-A, 45 (91.8) had successful pregnancy while 4 (9.2%) had miscarriage in comparison to 36 (73.5%) in Group-B had successful pregnancy whereas 13 (26.5%) had miscarriage and this difference was statistically significant in between the both study groups as women in Group-A had significantly better outcome in terms of successful pregnancy. (p-value = 0.0164). A local study from Bannu^10^ found very similar results where they noted oral progesterone, given as 10mg twice a day, to be effective in 90% of the women with threatened miscarriage. That study was a comparison of oral progesterone versus vaginal progesterone, and the authors also concluded that oral progesterone was significantly more efficacious than vaginal progesterone.^10^ A study done by Yassaee F et al.^12^ also noted efficacy of progesterone in terms of stretching pregnancy beyond 20^th^ week to be noted among 84.9% women which is closer to what we noted in the present study.

It is well known that pro-inflammatory cytokines are linked with miscarriage while progesterone induced blocking factor (PIBF) have inhibitory effects on immune reaction and shifting of cytokines from type-1 to type-2 causing an increase in the production of cytokines type-2.^13^ Pregnancy is commonly halted because of immunological factors, luteinic and neuroendocrine deficits as well as myometrial hypercontractility.^14^ All this may be helpful in explaining the low abortion rates among women given prophylactic progesterone.^15^

A local survey done in ordinary areas of Karachi by Ayub M et al.^16^ highlighted that women treated with progesterone, had reduced rates of abortion. Wang XX et al.^17^ noted oral progesterone to lower the risk of miscarriage (RR 0.55, 95% CI: 0.28-1.21) in pooled data analyzing data from 8 RCTs comprising of 845 women. Researchers have also indicated that women having low levels of progesterone (less than 35 mmol/L) have 24 fold more chances (OR 23.8, 95% CI 6.5-86.6%, p value < 0.0001) of miscarriage in comparison to those pregnant women having high levels of progesterone (equal or above 35 mmol/L).^18^

A Cochrane review done by Wahabi HA et al.^9^ reported that progestogens were found to minimize the risk of miscarriage in comparison to placebo or no treatment. That review also stated oral progesterone to probably reduce the risk of miscarriage but the researchers did not find any conclusive evidence about congenital abnormalities or preterm birth.^9^

The literature also reports some findings were inconclusive data about the benefits of progestogens was found in the treatment of threatened miscarriage.^19^-^21^ More trials with sound methodologies, randomized in nature, involving large sample size, multiple centers, and also having focus on delivery outcomes will further enlighten us about the aspects of progesterone in the treatment of threatened miscarriage.

### Limitation of the study:

One of the limitations of the current stud was that we could not record possible adverse outcomes related to progesterone treatment. Secondly, we also did not note fetal outcomes at the time of delivery in the selected women.

## CONCLUSION

The use of oral micronized progesterone was found to be significantly more effective than vaginal progesterone in women with threatened miscarriage. More randomized controlled trials are proposed to further verify the findings of this study.

### Author’s Contribution:

**RP:** Conceived, Data Collection and Responsible for data’s integrity and authenticity.

**MK:** Supervision and Proof Reading.

**ST:** Literature Review and Drafting.

**SM:** Data Collection and Data Analysis.
